# The roles of housing, financial, and food insecurities in understanding the relationship between childhood neglect and violence in adulthood

**DOI:** 10.1371/journal.pone.0246682

**Published:** 2021-03-03

**Authors:** Amie M. Schuck, Cathy Spatz Widom

**Affiliations:** 1 Department of Criminology, Law and Justice, University of Illinois at Chicago, Chicago, Illinois, United Sates of America; 2 Psychology Department, John Jay College and the Graduate Center, City University of New York, New York City, New York, United States of America; Brunel University London, UNITED KINGDOM

## Abstract

The aim of this study is to determine whether basic housing, financial, and food insecurities in part explain the relationship between childhood neglect and violence as documented in the “cycle of violence” literature. Using a prospective cohort design, neglected children (under the age of 12) with court substantiated histories (1967–1971) in one metropolitan Midwest area and demographically matched non-neglected children were followed into adulthood. Housing, financial, and food insecurities were assessed in 2003–2005 interviews at mean age 41. Official arrest data were used to measure violence ever and from 2003 through 2013. Mediation was tested using probit structural equation modeling. Controlling for age, sex, and race, childhood neglect predicted violent arrests and housing, financial, and food insecurities in middle adulthood. Housing and financial securities predicted violent arrests ever and after 2003, whereas food insecurity only predicted any violent arrest ever. Housing and financial insecurities partially mediated the relationship between childhood neglect and violent criminal behavior. Greater attention and efforts need to focus on providing basic housing, financial, and food support for neglected children to reduce their risk for violent criminal behavior.

## Introduction

The “cycle of violence” hypothesis predicts that individuals who experience physical abuse in childhood are at an increased risk for committing acts of violence in adolescence and adulthood [1]. A growing number of studies indicate that child neglect, defined as the lack of adequate food, clothing, shelter, and medical attention as a child, has a similar or larger effect on subsequent antisocial and violent behavior as physical abuse [2–4]. However, much less is known about the role of child neglect as a contributor to violent and criminal behavior compared to physical or sexual abuse, due to the “neglect of neglect” in research on child maltreatment [5]. Neglect is by far the most common form of child maltreatment and is linked to changes in the brain and nervous system, cognitive delays and impairment, and decreases in executive functioning [6]. In fiscal year 2018, 3.5 million children were referred for investigation of maltreatment and, of these, 678,000 children were determined to be victims of child maltreatment in the United States [7]. Three-fifths (60.8%) of these children were neglected only.

Housing, food, and financial insecurities in the United States are extensive. In 2018, about 37.2 million people lived in households with low food security and 9.5 million lived in households with very low food security [8]. Although there is no standard definition for housing insecurity, it typically includes overcrowding, frequent moves, staying with relatives or friends, difficulty paying rent, mortgage or utilities, and spending a significant amount of income on housing costs [9]. Based on the American Housing Survey (AHS), conducted by the U.S. Census Bureau every two years, in 2017, it was estimated that 37.8 million households paid more than 30% of income for housing and 18.2 million paid more than 50% [10]. As one indicator of financial insecurity, in 2018, there were 38.1 million people in poverty based on statistics from the U.S. Census Bureau [11].

Together, these insecurities characterize a condition of major deprivation where physiological and safety needs are not met. We draw on Maslow’s hierarchy of needs and apply this general framework to understanding the consequences of childhood maltreatment on later development [12]. We argue that Maslow’s theory may help explain why individuals who experience childhood neglect are at increased risk for later criminal and violent criminal behavior. Maslow outlined five interdependent levels of human needs that must be met for an individual to thrive and live a fulfilling life [13]. At the bottom are physiological needs (food, water, shelter, sleep and reproduction), followed by safety-security needs (security, stability, resources, and a lack of fear), belonging needs (friendship, love, family, and a sense of connection) and esteem needs (respect, status, recognition and self-esteem). At the top of the hierarchy are needs related to self-actualization, which Maslow defined as one becoming everything that he or she is capable of being. Maslow contended that people function at lower levels until those needs are met, at which time they can turn their attention to next higher level of needs.

Research suggests that there are key periods in childhood during which neurobiological systems are sensitive to specific experiences, and these time sensitive experiences affect structural and functional brain development [14]. By definition, neglected children are deprived of their basic needs for physical well-being and safety. Because victims of childhood neglect do not have their basic human needs met, they may miss important time sensitive experiences, and neurobiological and social development may be delayed, stunted or atypical. Research suggests that missed developmental periods resulting from environmental deprivation have the potential for long-term effects, especially in the areas of cognitive functioning and social attachment [15].

### Basic needs insecurities

A small, but growing, number of studies suggest that food insecurity is associated with an increased risk for antisocial behavior in adolescence and violence in adulthood [16–19]. Food insecurity and low-quality food may lead to later offending by negatively affecting cognitive functioning and self-control, raising stress levels, intensifying unhealthy conflict between partners, and contributing to mental health problems such as depression, anxiety and substance abuse [18]. The importance of housing goes beyond the physical structure that provides protection from environmental elements. Adequate housing is also related to the psychological and social well-being of residents [20]. While most of the research on housing insecurity focuses on physical health outcomes, mental health or the psychological benefits of adequate housing, there is some evidence that housing insecurity is associated with crime [17, 21, 22]. For example, in a qualitative study of women victims, housing instability along with food insecurity were identified as key contributors to intimate partner violence [17]. Research suggests that rates of violence are highest among those living in poor households [23] and in disadvantaged communities [24]. While the association between poverty and violence is generally acknowledged, controversy remains regarding causality. Not only is the causal direction under question, but some argue that the relationship is spurious with both being caused by larger social structures [25]. We hypothesize that food, housing, and financial insecurities representing unmet basic needs explain, in part, the relationship between childhood neglect and later violent criminal behavior.

## Method

### Design and participants

The data used here are from Widom’s (1989) large prospective cohort design study in which abused and/or neglected children (under the age of 12) with court substantiated histories of maltreatment between 1967 to 1971 in one metropolitan Midwest area were matched with non-abused and non-neglected children and followed into adulthood [26]. Abused and/or neglected children were matched with control children on age, sex, race/ethnicity and approximate family social class, which included neighborhood residence.

A non-maltreated, demographically matched control group was an essential element of original the study design. Elementary school records were used to find matches for school-age children (same sex, race and date of birth ± 6 months) and hospital birth records were used to find matches for children under school age (same sex, race, and date of birth ± 1 week). Matches were found for 74% of the maltreated children. When random assignment is not possible, Shadish, Cook, and Campbell (2002) recommend using hospitals and neighborhoods to match on measures that are associated with relevant outcomes [27]. Because a matching procedure was used, it is assumed that participants only differ in terms of the risk factor, that is, experiencing childhood abuse and/or neglect. However, the assumption of equivalency is an approximation because it was not possible to randomly assign participants to groups [26].

In the first phase of Widom’s (1989) study, the participants, both abused and/or neglected and matched controls, were compared on juvenile and adult criminal arrest records (*N* = 1,575). The second phase of the study involved locating and interviewing both groups during 1989–1995, approximately 22 years after the reported abuse or neglect (*N* = 1,196). Subsequent follow-up interviews were conducted in 2000–2002 (*N* = 896) and in 2003–2005 (*N* = 807). While there was attrition of participants over the various phases of the study due to deaths, refusals, and the inability to locate individuals, the composition of the sample has remained about the same over the waves of data collection. There were no significant differences across the phases of the study regarding sex, race, age or group membership (abused and/or neglected and control).

For this study, the analyses were restricted to participants in the 2003–2005 interviews and included only neglected individuals and matched controls (*N* = 713), of whom 51.3% (*n* = 366) were neglected and 48.7% (*n* = 347) were controls. Slightly more than half of the sample (51.5%) is female and 59% of the sample self-identified as White, non-Hispanic. All others included individuals who identified as Black, Hispanic, American Indian, or Pacific Islander. The average age of the respondents at the time of the interview during which the participants were asked about basic needs was 41.1 years old (*SD* = 3.5).

### Procedures

Interviewers were blind to the purpose of the study and to whether participants were neglected or not. Participants were also blind to the purpose of the study and were told that they were part of a study of individuals who grew up in the Midwest during the late 1960s and early 1970s. All participants were asked to sign a consent form indicating they were voluntarily participating. For individuals with reading problems, the consent form was presented and explained verbally. This study was approved by the Human Research Protection Program at The City University of New York (Protocol #: 2015–0133).

### Measures

#### Childhood neglect

Documented cases of childhood neglect were based on examination of family court records processed during the years 1967 to 1971 in a metropolitan county area in the Midwest part of the United States. Children were under the age of 12 years old at the time of the neglect experience. Neglect cases reflected a judgment that the parents’ deficiencies in childcare were beyond those found acceptable by community and professional standards at the time and represented extreme failure to provide adequate food, clothing, shelter, and medical attention to children. The neglect variable was coded 1 = an official court case of neglect, and 0 = matched control with no official documentation of neglect.

#### Insecurities

During the 2003–2005 interviews, participants were asked seven questions about food, housing and financial conditions during the prior 12 months. Four questions focused on food insecurity and asked whether or not participants had cut the size of meals or skipped meal because there was not enough food, lost weight because of a lack of food, and ever not eaten because of a lack of money for food. Participants were also asked to describe the amount of food the immediate family had in the past month using three potential responses (always enough to eat, sometimes not enough to eat, or often not enough to eat). These four questions were summed to create a food insecurity measure. Two questions focused on housing insecurity. Participants were asked if they could not afford a place to stay, and if they did not have a working telephone for two weeks or more. The two measures were summed to create the housing insecurity measure. One question focused on financial insecurity. Participants were asked about the family finances at the end of the month, whether they had some money left over, just enough money to make ends meet, or no money. For all three measures higher values indicated more insecurity.

Exploratory and confirmatory factor analyses indicated that the variables do not load on one latent factor, and as a consequence, mediation tests were conducted separately for each insecurity measure.

#### Violent criminal behavior

Information about whether the person had been arrested for a violent crime was based on the collection of complete criminal histories for these individuals at three levels of law enforcement (local, state, and federal) at three points in time (1986–1987, 1994, and 2012) [26, 28]. Violent criminal behavior included arrests for murder/attempted murder, manslaughter/involuntary manslaughter, reckless homicide, rape, sodomy, robbery/robbery with injury, assault, assault and battery, aggravated assault, and battery/battery with injury. We report any violent arrest through 2012 (ever) and any arrest for a violent crime between 2003 and 2012. Because the majority of the interviews were completed in 2003, this time period was selected to allow us to determine whether the violent crimes occurred after the information on insecurities was collected. At the time of that interview, the participants were middle aged (*M* = 41 years old) and past the peak years for violent offending [29, 30].

#### Control variables

Sex was coded 0 = male and 1 = female. To determine race and ethnicity, participants were shown a card with the names of racial and ethnic groups and asked to indicate which best described them. Race was coded 1 = White, non-Hispanic and 0 = all others. Age (in years) at the time of the interview was also included in the analyses.

### Statistical analysis

All analyses were conducted using the statistical software R [31]. Bivariate t-tests were used to investigate whether there were significant differences in food, housing and financial insecurities in young adulthood between the neglected and matched control participants. Because the homogeneity of variance test suggested that it was not appropriate to assume that the variances were equal, variances were estimated separately for each group using bootstrapping and the Welch modification. Mediation was tested using probit structural equation models, which included controls for sex, race, and age. Because of missing data, full information maximum likelihood (FIML) was used to estimate the path coefficients, which uses information from all available data. Fit indices were examined to determine goodness of fit, including chi square p-value > 0.05, comparative fit index (CFI) > 0.95, and root mean square error of approximation (RMSEA) ≤ 0.05).

## Results

Individuals with documented histories of childhood neglect reported significantly higher food, housing and financial insecurities than matched controls (see Table 1). Individuals with histories of neglect were also more likely than the controls to have ever been arrested for a violent offense and to have been arrested for a violent crime between 2003 and 2012. There were no significant differences between the two groups in terms of sex, race, or age.

**Table 1:** Descriptive statistics for the neglected and control groups.

	Total (*N* = 713)	Control (*n* = 347)	Neglect (*n* = 366)	*p*-value
Female (n, %)	367 (51.5%)	177 (51%)	190 (51.9%)	0.868
White, non-Hispanic (n, %)	421 (59%)	209 (60.2%)	212 (57.9%)	0.582
Age at interview (M, SD)	41.07 (3.5)	41.08 (3.6)	41.07 (3.5)	0.946
Financial insecurity (M, SD)	0.65 (0.03)	0.54 (0.04)	0.74 (0.04)	<0.001
Housing insecurity (M, SD)	0.31 (0.02)	0.25 (0.03)	0.37 (0.03)	0.005
Food insecurity (M, SD)	0.36 (0.03)	0.27 (0.04)	0.45 (0.04)	0.018
Any violent arrest (n, %)	167 (23.4%)	69 (19.9%)	98 (26.8%)	0.037
Any violent arrest 2003–2012 (n, %)	60 (8.4%)	18 (5.2%)	42 (11.5%)	0.004

Note: Comparisons of means and standard deviations were based on bivariate t-tests, whereas chi-square tests were used to compare percentages.

The results for the mediation models are presented in Tables 2–4. The models showed acceptable fit to the observed data, with nonsignificant chi-square values and CFI and RMSEA values in acceptable ranges. Table 2 shows significant direct paths from neglect to housing insecurity and from housing insecurity to any violent arrest and a significant indirect path from neglect to housing insecurity to any violent arrest. Table 2 shows a different set of relationships for the shorter time period (2003–2012) where there were significant direct paths from neglect to any violent arrest, neglect to housing insecurity, and housing insecurity to violent arrest, and a significant indirect path from neglect to housing insecurity to any violent arrest during the shorter time period. For the model in Table 2 the *R*^*2*^ was 0.01 for housing insecurity, 0.25 for any violent arrest after 2003, and 0.32 for any violent arrest ever.

**Table 2:** Results for analyses testing whether housing insecurity mediates the relationship between childhood neglect and violent criminal behavior.

	Any Violent Arrest (ever)			Any Violent Arrest (2003–2012)		
	Unstandardized Coefficient	Standard Error	95% Confidence Interval	Unstandardized Coefficient	Standard Error	95% Confidence Interval
Direct						
Neglect → Any violent arrest	0.21	0.11	[-0.01, 0.43]	0.40*	0.15	[0.11, 0.69]
Neglect → Housing insecurity	0.12*	0.04	[0.03, 0.20]	0.12*	0.04	[0.03, 0.20]
Housing insecurity → Any violent arrest	0.34***	0.07	[0.20, 0.49]	0.36***	0.10	[0.17, 0.55]
Indirect						
Neglect → housing insecurity → any violent arrest	0.04*	0.02	[0.01, 0.07]	0.04*	0.02	[0.01, 0.08]
Fit Indices						
Root mean square error of approximation	0.000			0.000		
Comparative fit index	1.000			1.000		
Chi-square	2.555			2.555		

Note: Analyses control for age, sex, and race.

* *p* < .05,

*** *p* < 0.001.

**Table 3:** Results for analyses testing whether financial insecurity mediates the relationship between childhood neglect and violent criminal behavior.

	Any Violent Arrest (Ever)			Any Violent Arrest (2003–2012)		
	Unstandardized Coefficient	Standard Error	95% Confidence Interval	Unstandardized Coefficient	Standard Error	95% Confidence Interval
Direct						
Neglect → Any violent arrest	0.18	0.11	[-0.04, 0.40]	0.37*	0.15	[0.07, 0.66]
Neglect →Financial insecurity	0.20***	0.05	[0.09, 0.30]	0.20***	0.05	[0.09, 0.30]
Financial insecurity → Any violent arrest	0.36***	0.07	[0.22, 0.49]	0.34***	0.08	[0.18, 0.50]
Indirect						
Neglect → financial insecurity → any violent arrest	0.07**	0.02	[0.02, 0.12]	0.07*	0.02	[0.02, 0.11]
Fit Indices						
Root mean square error of approximation	0.019			0.019		
Comparative fit index	0.964			0.947		
Chi-square	3.752			3.752		

Note: Analyses control for age, sex, and race.

* *p* < .05,

** *p* < 0.01,

*** *p* < 0.001.

**Table 4:** Results for analyses testing whether food insecurity mediates the relationship between childhood neglect and violent criminal behavior.

	Any Violent Arrest (Ever)			Any Violent Arrest (2003–2012)		
	Unstandardized Coefficient	Standard Error	95% Confidence Interval	Unstandardized Coefficient	Standard Error	95% Confidence Interval
Direct						
Neglect → Any violent arrest	0.22	0.11	[0.00, 0.44]	0.42***	0.15	[0.13, 0.71]
Neglect → Food insecurity	0.18*	0.08	[0.02, 0.35]	0.18*	0.08	[0.02, 0.35]
Food insecurity → Any violent arrest	0.16***	0.05	[0.06, 0.26]	0.11	0.06	[-0.01, 0.22]
Indirect						
Neglect → food insecurity → any violent arrest	0.03	0.02	[0.00, 0.06]	0.02	0.01	[-0.01, 0.05]
Fit Indices						
Root mean square error of approximation	0.000			0.000		
Comparative fit index	1.000			1.000		
Chi-square	1.776			1.776		

Note: Analyses control for age, sex, and race

* *p* < .05,

*** *p* < 0.001.

Table 3 shows the results for financial insecurities. Similar to the results for housing insecurity, there were significant direct paths from childhood neglect to financial insecurity, from financial insecurity to any violent arrest (ever), and a significant indirect path from childhood neglect to financial insecurity to any violent arrest (ever). For the shorter time period (2003–2012), there were significant direct paths from neglect to violent arrest, neglect to financial insecurity, and financial insecurity to violent arrest, and a significant indirect path from neglect to financial insecurity to any violent arrest. For the model in Table 3 the *R*^*2*^ was 0.02 for finance insecurity, 0.25 for any violent arrest after 2003, and 0.34 for any violent arrest ever.

Finally, Table 4 shows a different set of results for the mediation analyses testing whether childhood neglect leads to violent criminal behavior through food insecurity. There were significant direct paths from neglect to food insecurity and from food insecurity to any violent arrest (ever), but no significant indirect path. For the shorter time period (2003–2012), there were significant direct paths from neglect to violent arrest and from neglect to food insecurity, but no significant direct path from food insecurity to violent arrest and no significant indirect path from neglect to violent arrest through food insecurity. These two sets of findings (ever and the shorter time period) do not provide support for the hypothesis that food insecurity mediates the relationship between childhood neglect and later violent behavior, although food insecurity is associated with ever being arrested for a violent crime. For the model in Table 4 the *R*^*2*^ was 0.01 for food insecurity, 0.22 for any violent arrest after 2003, and 0.31 for any violent arrest ever.

## Discussion

In this prospective cohort design study that followed individuals with court substantiated cases of neglect and matched controls into adulthood, we found that childhood neglect predicted violent arrests and housing, financial, and food insecurities in middle adulthood more than 30 years later. These new results also show that housing and financial insecurities predicted ever being arrested for a violent crime and being arrested for a violent crime during the shorter 10-year time period after 2003 in middle adulthood. As hypothesized, housing and financial insecurities partially mediated the relationship between childhood neglect and subsequent violent offending. This finding held even after restricting the violent criminal behavior to the time after the in-person interviews were conducted (between 2003 and 2012). A different pattern of results emerged for food insecurity. While childhood neglect predicted food insecurity and food insecurity predicted any violent arrest (ever), we did not find food insecurity predicted violence in the shorter time period in middle adulthood or that food insecurities mediated the relationship between childhood neglect and violence.

These results provide new evidence of the impact of these childhood experiences into middle adulthood and show the role of housing, financial, and food insecurities in understanding the consequences of childhood neglect and the cycle of violence. These insecurities were not only related to the commission of violent crime ever, but also in middle adulthood when most violent offending has typically tapered off [29, 30]. While the amount of variance explained in the insecurities measures is small, it is important to point out that these basic needs insecurities were assessed approximately 30 years after the childhood neglect in adulthood and information was obtained only about past year insecurities. This research raises new questions about the mechanisms that may underlie these relationships. For example, it is possible that these basic needs insecurities place previously neglected individuals at increased risk for violence by raising stress levels, reducing feelings of control, and/or increasing feelings of anger, resentment, and alienation, which in turn, serve to trigger antisocial behavior and violent offending. It is also possible that these insecurities lead to uncertainty about one’s ability to survive in the world. Research suggests that uncertainty can magnify negative events by intensifying an individual’s affective reactions to the event [32].

We found that childhood neglect predicted food insecurity in middle adulthood and that food insecurity predicted an increased risk of arrest for a violent crime ever. However, we did not find that food insecurity was a significant mediator of the relationship between childhood neglect and violence. This was surprising, particularly since basic food needs are at the first (lowest) level of Maslow’s hierarchy of needs and were expected to play a major role in understanding violence. Several studies have connected food insecurity to intimate partner violence [16, 18]. However, these studies have all focused on domestic violence, not any violence. In one prospective longitudinal study, Belsky et al. (2010) found that the association between food insecurity and behavioral problems disappeared after taking into account the characteristics of the mother including depression, poor self-control and antisocial tendencies [33].

Prior research has suggested that neglect is related to physiological changes, reductions in executive functioning and self-control, the development of maladaptive coping strategies, mental health problems and substance abuse, [6, 12] all of which may reduce a person’s ability to function effectively in adulthood. It is also possible that feelings of insecurity may result in more biased decision making and less thought of the future and greater effort spent acquiring resources at the expense of a longer term perspective [34]. If individuals with histories of neglect focus their efforts on meeting basic needs, this may come at the expense of finishing high school, getting a college degree, or finding adequate employment. There is some support for this proposition in the research that has linked childhood maltreatment to lower levels of academic achievement, unstable employment, and residence in neighborhoods in adulthood with more economic disadvantage [35, 36].

Finally, these results reveal the importance of housing insecurities in relation to violent criminal behavior. The role of stable housing may be critical when providing services to victims of neglect. Some service providers are advocating for “housing first” programs, based on the assumption that unless an individual has a home, he or she does not have an adequate base or platform to successfully address other challenges in his or her life such as addiction, psychiatric symptoms, or employment [20, 37]. These new findings would lend support to such efforts.

Despite the many strengths of this study, limitations should be noted. These results are based on a large sample of children with documented histories of childhood neglect and a matched control group, both of whom grew up in one Midwestern location during the late 1960s and early 1970s. The sample is skewed toward lower socioeconomic status and, thus, caution should be used in generalizing from these findings to neglected children from middle- and upper-class families. However, these findings are particularly meaningful because the neglected children and controls were matched on approximate family social class and were from the same childhood neighborhoods and, therefore, these relationships are unlikely to be explained by poverty in childhood. While there were no known cases of childhood maltreatment among the controls, it is possible that some controls experienced neglect that did not come to the attention of authorities (false negatives). However, in general, the presence of false negatives in the control group should reduce the likelihood of finding statistically significant differences between the two groups. The limitations of reliance on official criminal records apply [28]. Finally, the measures used to assess insecurities regarding food, housing and finances were limited and future work would benefit from a more comprehensive assessment. In particular, the comparative fit index values for the financial insecurities models were right at the cut off for a good fitting model indicating that this measure would benefit from greater specification.

## Conclusions

While attention has typically focused on childhood physical abuse in studies of the “cycle of violence”, this research has highlighted the importance of childhood neglect in the development of antisocial and violent behavior. Using a longitudinal design with documented cases of childhood neglect, the current research shows that housing and financial insecurities in adulthood partially explain the relationship between childhood neglect and later violent criminal behavior. This work has implications for programs and interventions that should ensure that individuals who have been neglected have their basic needs met and represents a first step in understanding the role of these basic needs insecurities on development.
